# Dental MRI using wireless intraoral coils

**DOI:** 10.1038/srep23301

**Published:** 2016-03-29

**Authors:** Ute Ludwig, Anne-Katrin Eisenbeiss, Christian Scheifele, Katja Nelson, Michael Bock, Jürgen Hennig, Dominik von Elverfeldt, Olga Herdt, Tabea Flügge, Jan-Bernd Hövener

**Affiliations:** 1 Medical Physics, Department of Radiology, University Medical Center Freiburg, Freiburg, Germany; 2 Division of Oral and Maxillofacial Surgery, University Medical Center Freiburg, Freiburg, Germany

## Abstract

Currently, the gold standard for dental imaging is projection radiography or cone-beam computed tomography (CBCT). These methods are fast and cost-efficient, but exhibit poor soft tissue contrast and expose the patient to ionizing radiation (X-rays). The need for an alternative imaging modality e.g. for soft tissue management has stimulated a rising interest in dental magnetic resonance imaging (MRI) which provides superior soft tissue contrast. Compared to X-ray imaging, however, so far the spatial resolution of MRI is lower and the scan time is longer. In this contribution, we describe wireless, inductively-coupled intraoral coils whose local sensitivity enables high resolution MRI of dental soft tissue. In comparison to CBCT, a similar image quality with complementary contrast was obtained *ex vivo*. *In-vivo*, a voxel size of the order of 250∙250∙500 μm^3^ was achieved in 4 min only. Compared to dental MRI acquired with clinical equipment, the quality of the images was superior in the sensitive volume of the coils and is expected to improve the planning of interventions and monitoring thereafter. This method may enable a more accurate dental diagnosis and avoid unnecessary interventions, improving patient welfare and bringing MRI a step closer to becoming a radiation-free alternative for dental imaging.

Tomographic imaging of hard- and soft-tissue is of great interest in oral surgery and dental implantology for example to evaluate bone volume, gingiva and vital structures in order to plan and perform surgical interventions. Unfortunately, neither the X-ray based gold standard, computed tomography (CT) and cone-beam CT (CBCT), nor magnetic resonance imaging (MRI) provide soft and hard tissue imaging simultaneously.

Only recently, however, MRI sequences with very short echo time (TE) were reported that enabled fast soft- and hard-tissue imaging of hydrogen and other nuclei like sodium^1^,^2^,^3^,^4^,^5^,^6^,^7^,^8^. This development stimulated a rising interest in dental MRI^9^,^10^,^11^,^12^,^13^,^14^,^15^, and detailed images with soft- and hard-tissue contrast of extracted human teeth were already obtained *ex vivo:* Using a small-bore MRI system at high field in conjunction with ultra-short-TE (UTE), zero-TE (ZTE), sweep imaging with Fourier transformation (SWIFT) or single-point imaging (SPI), a voxel size of the order of 100 μm^3^ was achieved.

*In vivo*, however, these results are not yet reproduced. This is because conventional MRI sequences like *fast low angle shot* (FLASH)^16^ or *rapid acquisition with relaxation enhancement* (RARE)^17^ cannot capture the rapidly decaying MR signals of solids like enamel or compact bone because TE is too long (typically a few ms).

UTE, SWIFT and ZTE sequences are not widely available for clinical MRI systems yet, but promising proofs of principle were already reported^10^,^15^,^18^,^19^,^20^. Another reason is the field of view (FOV), which is much larger *in vivo* (typically the entire head) than for a single tooth^11^. For a typical MRI examination, several minutes are required to provide images with a voxel size of the order of 1 mm^3^ that have great soft tissue contrast but no hard tissue signal^21^.

As a result, MRI is hardly employed for routine dental imaging, but X-ray based methods are used instead. Within seconds, CT and CBCT provide high-resolution images with great hard tissue contrast, however, almost no soft-tissue signal. It must be kept in mind, though, that CBCT and CT exposes the patient to harmful ionizing radiation, which should be kept at a minimum (ALARA principle): even though radiation doses in dental exams are small, an increased cancer incidence has been found in patients^22^,^23^ and medical personnel^24^ as a consequence of old X-ray systems and repetitive exams.

It was recognized before that the low sensitivity of MRI is a major hindrance for high-resolution imaging of soft- and hard-tissue. Whereas hyperpolarization methods^25^,^26^,^27^,^28^,^29^,^30^,^31^,^32^,^33^ promise to mitigate this limitation for metabolic investigations (spectroscopy)^34^ and lung imaging, these methods aren’t applicable to anatomical imaging yet. Another approach to improve the sensitivity is using specialized coils. At least three dedicated, cable-bound surface coils have been developed to overcome the low filling factor of dental MRI with conventional head- and neck imaging coils^10^,^15^,^18^, one of which is commercially available (4-channel dental-array, Noras MRI Products GmbH, Höchberg, Germany)^35^. The sensitive volume of these coils is of the order of 1000 cm^3^ and was designed to allow concurrent imaging of many teeth with a voxel size of the order of (350–800 μm)^3^ 15,18,35,36.

In this work, we propose to use wireless, inductively-coupled intraoral volume coils for dental MRI whose local sensitivity, i.e. sensitive volume, is of the order of few cm^3^ only, much smaller with respect to previous implementations. We hypothesize that these coils will enable dental MRI in humans with an improved resolution within clinically feasible scan times.

## Methods

### Construction of Coils

**Figure 1:**
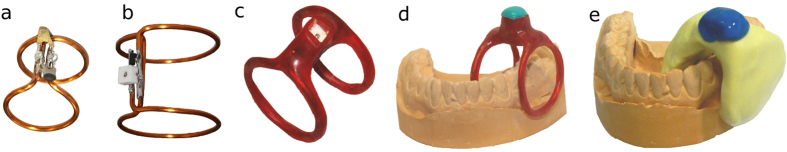
Photographs of wireless, inductively-coupled intraoral coils: bare C_1_-type coil with fixed capacitors (**a**), C_3_-type with variable capacitor and diodes before (**b**) and after (**c**) coating with a dental resin, mounted on a mandible cast with sealed tuning channel (**d**) and ready for *in-vivo* use with silicon coating (**e**).

The coils consisted of two coaxial loops of 1.5 or 2 cm diameter, respectively, at a distance of 1.7 cm, wound from 1.25 mm diameter insulated copper wire (enameled copper wire, Part 1230986, Rowan Cable Products Ltd, UK) and adapted to human anatomy using a dental stone cast. The coils were tuned to the hydrogen resonance frequency at 3 tesla (123.24 MHz). Three variants of the coils were evaluated: In its simplest from, a fixed-value capacitor was soldered to the wire (coil type C_1_, diameter 1.5 cm, Fig. 1a); in a second variant (C_2_), crossed-diodes were added in parallel to the capacitor to provide passive detuning during RF excitation (1.5 cm diameter, BAV 99, MULTICOMP, Germany, or BAR64-04W, Infineon, Germany); in variant three (C_3_), a variable ceramic capacitor was placed in parallel to the crossed diodes to enable tuning of the coil for variable loads (Fig. 1b 2 cm diameter, 2.5–10 pF, 250 V, CERAtrim, Johanson Manufacturing, USA). After the construction, the coils were coated with an acrylic insulation spray (Plastik 70, CRC-Kontaktchemie, Germany).

For *in-vivo* application, the C_3_ coils were coated with two more layers of dental materials that are approved for *in-vivo* use: A dental acrylic resin (pattern, GC America Inc., USA) was applied and milled to yield a smooth surface after hardening, maintaining an access channel to the variable capacitor (Fig. 1c,d). Another layer of soft silicon putty (Optosil, Heraeus Kulzer, Germany) was added to improve the intraoral fixation of the coil and to reduce the sensitivity of the resonance frequency to motion of the tongue and the jaw (Fig. 1e). These layers ensured that no direct contact potentials were formed with adjacent tissue structures.

After placement *in vivo* or *ex vivo*, the resonance of C_3_ coils was adjusted using a network analyzer in S11 mode with a pick up coil (ZVB4 Vector Network Analyzer, Rhode & Schwarz, Germany). The access channel was sealed with silicon putty before the subject entered the magnet (Xantopren, Heraeus Kulzer, Germany).

### Quality factor Q and Inductivity L

The quality factor Q of the intraoral coils was measured by inductive coupling to a 8.5 cm diameter pick-up coil (i) without load, (ii) mounted a test tube (phantom P_1_) and (iii) *in vivo* (for C_3_ only, Table 1). P_1_ contained 21 mL of a solution made from 100 ml deionized water and 1 mL Gadopentetat-Dimeglumin solution (0.5 mMol/ml, Magnevist, Bayer, Germany). The reduction of Q that was observed *in vivo* is likely attributed to the coupling of the coil with the surrounding tissue. A similar effect was observed for the other coils when a hand was moved close to the coil. The inductivity L of the resonators was derived from the resonance equation *f* = 1/(2 ∙ π ∙ (LC)^½^) to approximately 150 nH.

### MRI

A standard 3 T whole-body MR system with transmit body coil was used (MAGNETOM Trio, A Tim System, upgraded during the study to a MAGNETOM PRISMA, Siemens Healthcare, Germany) in conjunction with either a standard 12-channel head matrix coil (Siemens Healthcare, Germany), four channel carotid artery coil (Rapid Biomedical, Germany) or single-channel loop coil (4 cm diameter, Siemens). No water or fat suppression schemes were used.

### Decoupling

To investigate the coupling of the intraoral coils with the body coil during transmission, two intraoral coils at a time (C_2_ and C_3_, C_1_ and C_3_) were mounted on phantom P_1_ that was placed on a cylinder filled with 2 L of an NiSO_4_-doped aqueous solution (phantom P_2_, 3,75 g NiSO_4_ · 6 H_2_O + 5 g NaCl per liter) in the isocenter of the magnet. The coils were mounted at opposite ends of P1 to avoid mutual coupling, but to enable quantitative evaluation on one image. The slice for the automated adjustments of frequency, flip angle, receiver gain and shim was set at a distance of approx. 10 cm from P_1_.

3D FLASH images were acquired at nominal flip angles of α = 1°–40° using non-selective excitation pulses (TR = 50 ms and TE = 3.5 ms). The signal was evaluated in four places: two regions within the intraoral coils, one in between (all in P_1_), and one in P_2_. The maximum flip angle allowed for the sequence was 40°.

### *Ex-vivo* studies

**Figure 2:**
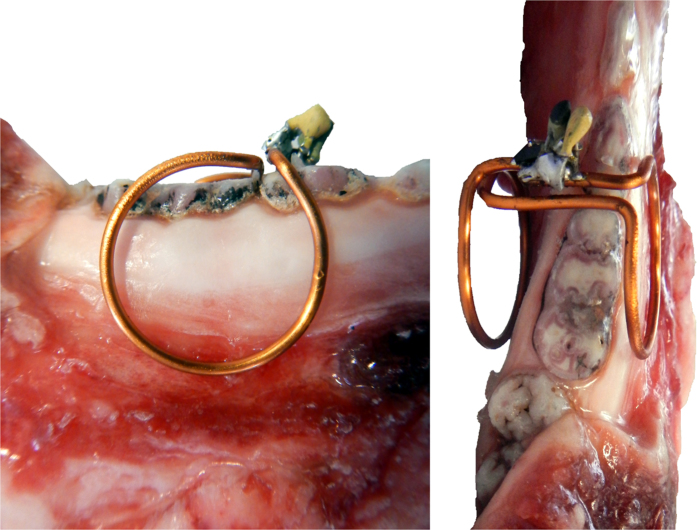
Typical placement of an intraoral coil (here: C_2_) on a porcine mandible for *ex-vivo* MRI as shown in Figs 5 and 6.

For *ex-vivo* studies, a fresh porcine mandible and a human mandible preparation conserved in 4% aqueous formaldehyde were used. Because of the coupling to the transmit coil, C_1_-type coils were mounted on the specimen after the sample was placed in the magnet and the adjustments of the MRI system were completed (Fig. 2). C_2_ and C_3_-type coils were mounted on the specimens and placed in the magnet together before the adjustments, a few centimeters away from its isocenter (where the adjustments of the MR system were performed).

### *In-vivo* studies

A 29 years old female subject was investigated using C_3_-type coils in conjunction with the head coil, the carotid-artery coil or the 4-cm loop coil. Imaging parameters are indicated where appropriate.

Images were processed using the manufacturer’s software (Siemens, Sordex, USA), IMPAX (AGFA Healthcare, Mortsel, Belgium) as well as imageJ (National Institutes of Health, USA)^37^, GIMP (www.gimp.org) and Inkscape (www.inkscape.org) to arrange images and add indicators. The signal to noise ratio (SNR) of a region was calculated by dividing the mean signal of the region by the standard deviation of the noise elsewhere, measured with imageJ. The error is estimated to be 10%.

### Cone beam computed tomography (CBCT)

For comparison a CBCT system (Scanora 3D, soredex, USA) for dental clinical routine was used to acquire images of the porcine jaw with a nominal resolution of (130 μm)^3^ in a (60 mm)^2^ FOV at 85 kVp.

### Ethics

*In-vivo* imaging and *ex-vivo* imaging of a dissected human mandible, provided by the Institute of Anatomy and Cell Biology, were carried out in accordance with the guidelines of application N. 338/13 of the Department of Oral and Maxillofacial Surgery approved by the Ethics Committee of the Albert-Ludwigs-University of Freiburg. Written informed consent was obtained from the subject prior to the MR examination.

## Results

### Decoupling

**Figure 3:**
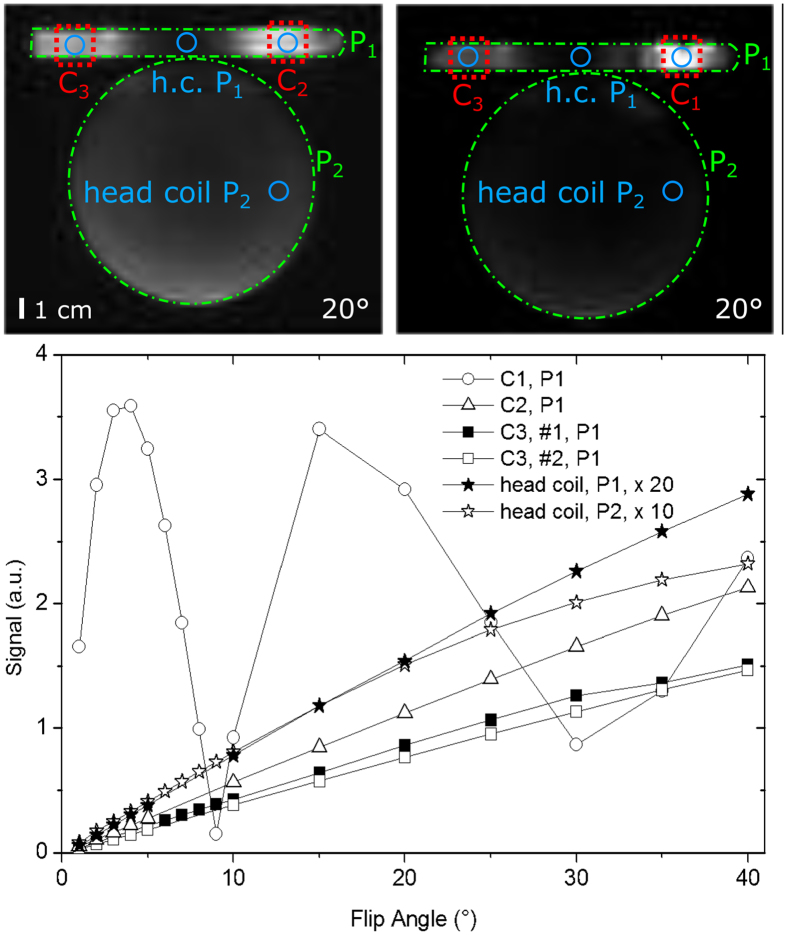
Single slice of a 3D ^1^H-FLASH MRI (top). The intraoral coils (C_1_, C_2_, C_3_), the phantoms (P_1_, P_2_) and the regions-of-interest (ROI) are indicated by dotted red, dotted-dashed green and solid blue lines, respectively. The signal intensities of the ROIs was measured and plotted as function of the flip angle (bottom). The signals from the decoupled coils C_2_, C_3_ (triangles and squares) exhibited a similar response like the signal from the head coil (stars), indicating an effective decoupling. Note that C_3_ was used twice, and that the head-coil-only signals from P_1_ and P_2_ were multiplied by a factor of 20 and 10 for better visualization, respectively.

The decoupling during excitation was investigated *ex vivo* by mounting the coils on a test tube (P_1_) as described above (Fig. 3, top). The signals from within C_2_, within C_3_ and in between C_2_ and C_3_ exhibited a similar response to the flip angle variation as the signal acquired with the head coil alone (Fig. 3, bottom). The fact that the signals within and in between C_2_ and C_3_ exhibited a similar response indicates that the decoupling was effective and that no uncontrolled, potentially harmful r.f. was generated within these coils.

At 40°, the highest flip angle allowed by the sequence, the signal maximum expected from the FLASH equation was not reached because the relaxation time T_1_ of P_1_ is short. At this angle, the signal from P_1_ within C_2_ was 2128 a.u. and 15 times higher than the signal from P_1_ in between C_2_ and C_3_ (144 a.u.); likewise, the signal from within C_3_ was 10 times higher at 1488 a.u. (the latter number is the mean of two measurements).

In contrast, the signal from within the non-decoupled coil C_1_ rose much faster and reached a maximum of 3588 a.u. at 5°. This response to the flip angle variation indicates a strong coupling between excitation and intraoral coil. For larger flip angles, the inhomogeneous excitation profile became visible within the (coupled) coil.

**Figure 4:**
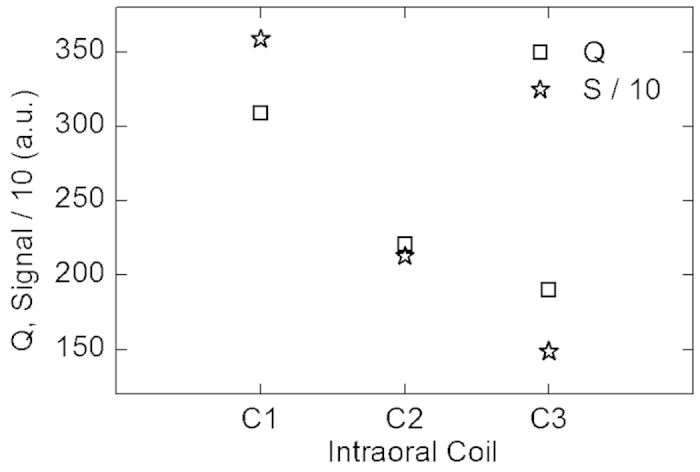
**Maximum**^1^H-FLASH MRI signal (stars) and Q (squares) of intraoral coils C_1_-C_3_ mounted on P_1_. For MRI, the coils were inductively coupled to the head coil (Fig. 3).

The maximum signal intensities from within all inductively-coupled coils (3588 a.u., 2128 a.u., 1488 a.u.) exhibited the same trend as Q that was measured on the same phantom (309, 221, 190, Fig. 4).

### *Ex-vivo* MRI with non-decoupled coils

**Figure 5:**
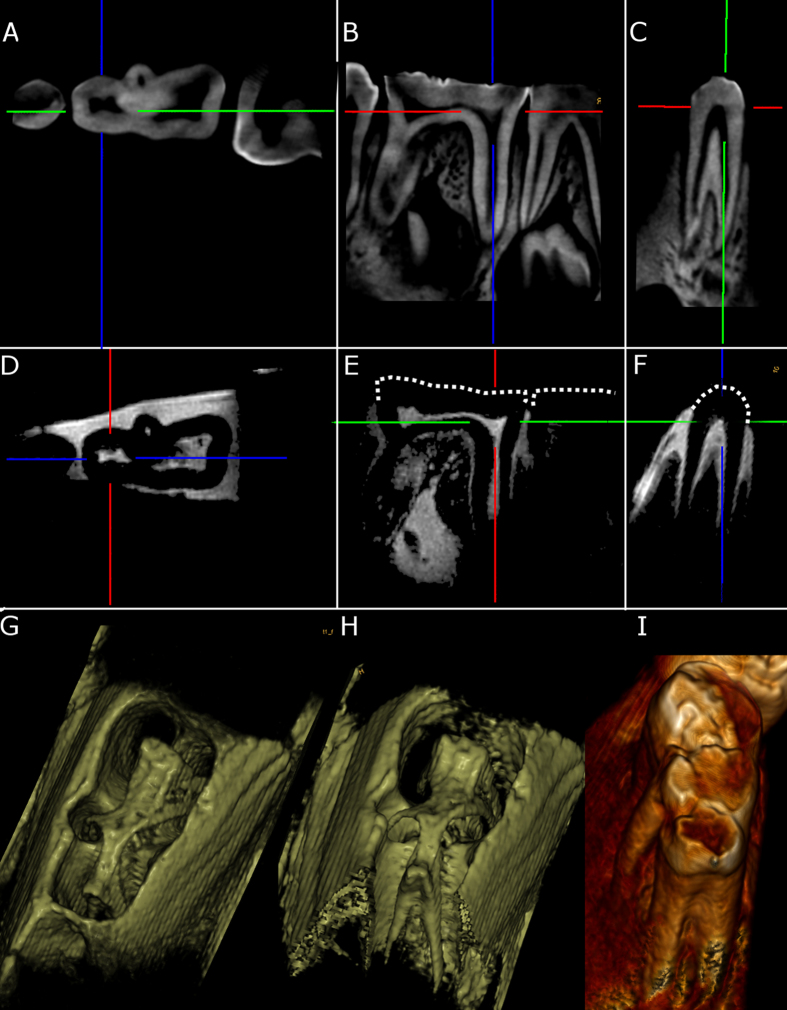
*Ex-vivo* dental CBCT and 3D-FLASH MRI acquired with coil C_1_ of a porcine mandible. Reconstructed orthogonal views and 3D rendering of CBCT (**A–C,I**) and MRI (**D–H**) with a voxel size of (130 μm)^3^ and (270 μm)^3^, respectively. A volume of approximately 30 cm^3^ was acquired five times in 5:32 min in conjunction with a head coil (TR/TE = 11/4.6 ms, 96 slices). Note the complementary signal of MRI and CBCT e.g. in gingiva and bone. The crowns of the teeth are indicated by dotted lines in (**E**) and (**F**). Colored lines indicate the slice positions.

The results above (Fig. 3) indicate that a homogeneous, accurate and controlled application of flip angle and r.f. power is challenging with non-decoupled coils of type C_1_. Still, these coils provide a higher Q and signal and may be advantageous for *ex-vivo,* gradient-echo MRI with a low flip angle, as demonstrated by imaging a porcine jaw with a 3D FLASH sequence with an isotropic voxel size of (270 μm)^3^ in 5:23 min (Fig. 5D–H, TR = 11 ms, TE = 4.6 ms, 96 slices, 128^2^ matrix, FOV 34∙34∙33.6 mm^3^ ≈30 cm^3^, bandwidth 190 Hz/px, maximum signal at nominal flip angle 1°, 5 averages).

The gingiva, dental pulp, periodontium and liquid-filled cavities in the cancellous bone of the specimen were well depicted.

In fact, these images appear comparable in resolution to the clinical gold standard, CBCT, which was acquired with a nominal isotropic resolution of 130 μm (Fig. 5A–C,I). In contrast to the MRI, however, the CBCT signal is rather complementary and originated mostly from enamel, dentin and cortical bone, as emphasized by 3D surface renderings of both datasets (Fig. 5G–I).

### *Ex-vivo* MRI with decoupled coils

**Figure 6:**
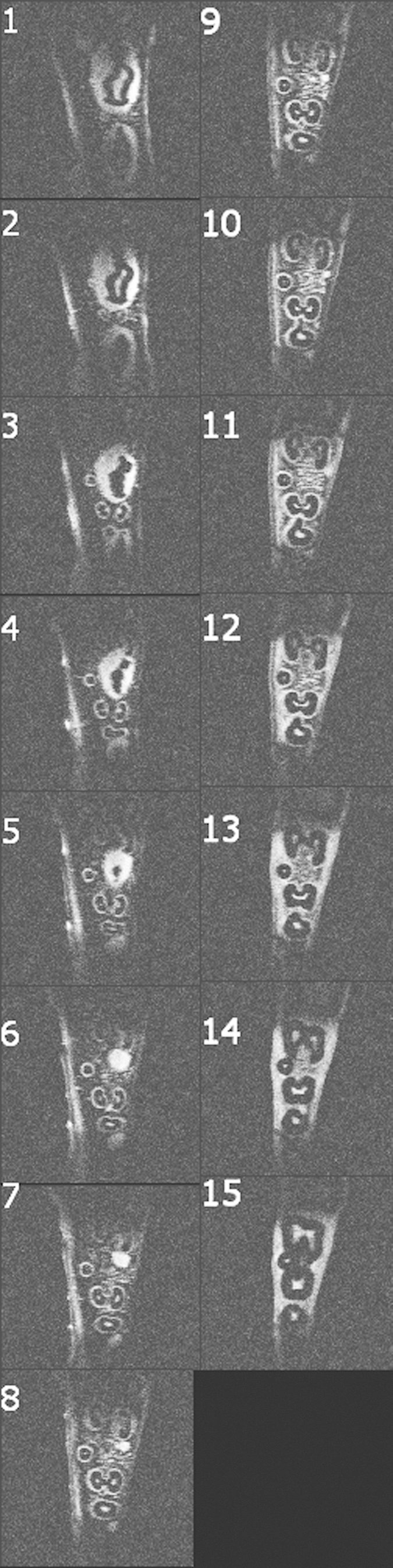
*Ex-vivo* 2D RARE MRI of a porcine jaw acquired with coil C_2_ in conjunction with a head coil. In 2:38 min, an in-plane voxel size of (300 μm)^2^ was achieved in 23 slices of 600 μm thickness (TR = 3.75 s, TE = 11 ms, 3 echoes, 900 μm slice distance).

In contrast to C_1_-type coils, the decoupled coils allowed to take advantage of the calibrated and homogeneous excitation pulses of the body resonator e.g. to apply a RARE^17^ sequence: in 2.38 min, 23 slices were acquired with a (300 μm)^2^ in-plane resolution and 600 μm thickness (Fig. 6, TR = 3.75 s, TE = 11 ms, 3 echoes, distance slice center-to-center 900 μm).

**Figure 7:**
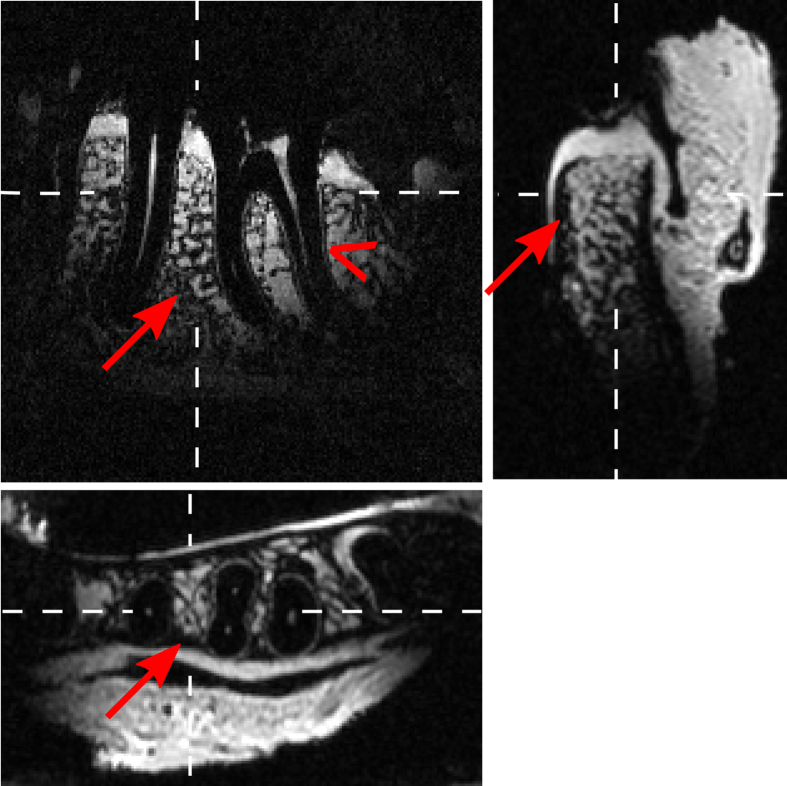
*Ex-vivo* 3D-FLASH MRI of a human mandible acquired with coil C_3_ in conjunction with a head coil in 4:28 min at 3 T. Reconstruction of orthogonal slices with an voxel size of 200∙200∙400 μm^3^ (TR = 12 ms, TE = 4.3 ms, two averages). Note the differentiation of cancellous and compact bone and, possibly, the periodontium as indicated by arrows and wedge, respectively.

We noted that the resonance frequency changed significantly up to 4 MHz as function of the coil’s loading, resulting in detuning. Minor adjustments of the resonance frequency of the order of 1 MHz were possible by bending the loops of coils, but a variable capacitor allowed a much better adjustment at the cost of some loss in Q. With this coil (C_3_), a human jaw specimen was imaged with two averages of a 3D FLASH sequence that had a voxel size of 200∙200∙400 μm^3^ in 4:28 min (Fig. 7, TR = 12 ms, TE = 4.3 ms, FOV = (39∙39∙24) mm^3^ = 36.5 cm^3^, 192^2^ matrix, 120 slices, α = 15°, slice resolution 50%, bw = 239 Hz/px)). The gingiva, marrow-filled cavities of the cancellous bone, compact bone (arrows), the dental pulp and a structure that may be assigned to the periodontium (wedge) were depicted.

### *In-vivo* MRI with decoupled coils

**Figure 8:**
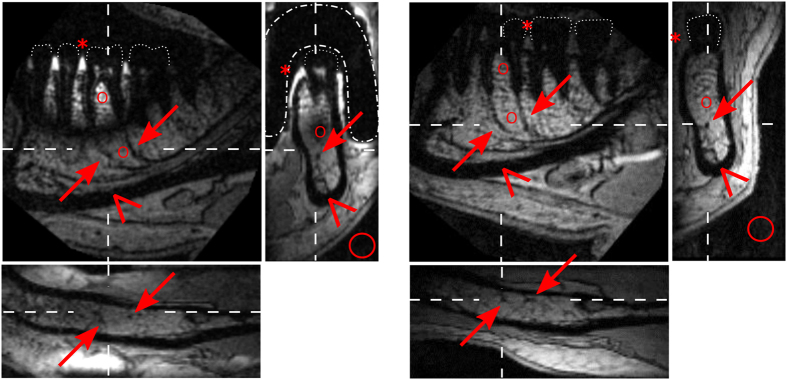
*In-vivo* dental MRI of a mandible acquired with (**a–c**) and without (**d–f**) intraoral coil C_3_ in conjunction with a 4-cm loop coil. Orthogonal slices were reconstructed from a 3D-FLASH MRI that was acquired in 3:57 min at 3 T at a resolution of 250∙250∙500 μm^3^. Note that the intraoral coil enhanced the signal of the interdental gingiva (asterisk) and pulp strongly and caused a signal hypointensity at the roots. Both configurations nicely depicted the inferior alveolar nerve and its branches to the apices of the teeth (rami dentales) as indicated by arrows and wedges, respectively. Dashed lines indicate the position of the reconstructed slices, dotted lines the crowns of the teeth, dash-dot lines the position of the intraoral coil. The regions where the signal and noise was measured are indicated by asterisks (gingiva) and circles, see Table 2.

Because effective decoupling was achieved (with the crossed diodes) and *in-situ* tuning was enabled, we were able to translate the presented coil concept *in vivo*. High-resolution 3D-FLASH MRI of a mandible was acquired (Fig. 8, left). In 3:57 min, a volume of 64∙64∙28 mm^3^ = 115 cm^3^ was imaged with a voxel size of 250∙250∙500 μm^3^ (Fig. 8, left, TR/TE = 11/4.2 ms, α = 15°, bw = 280 Hz/px, in conjunction with the 4-cm loop coil). Strong signal was observed from dental pulp and the tooth-surrounding gingiva. Both the inferior alveolar nerve and its branches to the teeth (rami dentales) were readily depicted. The signal void of the coil’s silicon coating facilitated the delineation of the alveolar rim and surrounding tissue. Note that a hypointensity was observed at the root apex, approximately in between the loops of the coil, which is attributed to the low sensitivity of the coil in this region.

For comparison, the intraoral coil was removed and the experiment was repeated with the same sequence parameters (Fig. 8, right). Whereas the pulp and gingiva yielded much less signal, no signal void was observed and the alveolar nerves were depicted completely.

In its sensitive volume, the SNR (31–57) of the intraoral coil in conjunction with the loop coil exceeded the SNR (2–16) of the loop coil alone up to 16 fold; outside of the sensitive volume, the SNR was comparable (19–20). Within the void of the intraoral coil, the SNR was close to the noise level, whereas the SNR of the loop coil was 17 (Table 2). As expected, the gain in SNR was strongest for the regions farthest away from the loop coil.

## Discussion

The quality of the images that were acquired *ex vivo* (Figs 4, 5, 6) and *in vivo* (Fig. 8) in a few minutes only suggests that the presented concept of using wireless, inductively-coupled intraoral receive coils is a viable approach to improve the performance of dental MRI. In the sensitive volume of the coil, the SNR was improved 7 to 16-fold. The coils are easily combined with any clinical MR system and standard sequences, and provide detailed information about 2–4 teeth and the surrounding tissues.

### Comparison to other MRI coils

When compared to other dental coil implementations of larger volumes described in literature^15^,^18^,^35^,^36^, the local sensitivity of the wireless intraoral coils provided a strongly improved resolution and display of relevant anatomical details. When compared to images acquired with the 4-cm loop coil alone (Fig. 8d–e), the interdental gingiva and dental pulp were much better depicted with an increase in SNR of 7 to 16 fold (Table 2). We note, though, that a signal hypointensity in between the loops of the intraoral coil was observed.

One advantage of both intraoral and loop coil is that the rami dentales of the inferior alveolar nerve are well depicted as compared to the published images acquired with other dental MR coils^15^,^18^,^35^, which is of diagnostic interest. The reconstruction of arbitrary slices is feasible for images acquired with an isotropic voxel size of (350 μm)^3^ (not shown) but also for non-isotropic voxels that yield a higher in plane resolution as shown in Fig. 8.

### Comparison with CBCT

With respect to the clinical gold standard, CBCT, the image quality appears comparable but complementary, although tested *ex vivo* only (Fig. 5). As expected, the soft-tissue contrast provided by the intraoral coils was superior and reaches CBCT resolution. The hard-tissue contrast is only indirect. This finding is attributed to the fact that the gradient echo and spin echo sequences used are not suitable to detect the fast decaying signal of hard tissue. SPI, SWIFT, UTE and ZTE sequences have demonstrated hard-tissue signal and may be explored with this coil in the future. It should be noted, though, that water-containing structures such as gingiva or pulp provide strong signal that can mask the weak signal of hard tissue like enamel or dentin and may require suppression schemes as described in ref. 20. A quantitative comparison of CBCT and intraoral MRI is underway and will be described elsewhere^38^.

### Prospective diagnostic application

CBCT is used in dentistry for diagnostic imaging with regard to various indications related to dental and bony hard tissues, however soft tissues are not displayed. With MRI, cancellous bone, intraoral mucosa, dental pulp and periodontium are directly displayed, whereas cortical bone and dental roots are indirectly displayed, by the contrast to surrounding soft tissues. It appears that MRI imaging is suitable for the majority of indications in which CBCT is currently used, given an adequate quality and practicability is achieved. Possible indications include dental implant therapy and preoperative planning, inflammatory diseases of the tooth, periodontium and alveolar bone and neoplastic processes.

### Properties of the coils described

Intrinsically, the imaging volume of the presented coils is limited and even smaller than the volume of other coils for dental MRI^15^,^18^. This property makes the presented approach suitable for the investigation of selected teeth prior to a surgical intervention for planning or afterwards to monitor the healing process after extraction or placement of a ceramic implant e.g. Detailed imaging of bone defects like necrosis or inflammation that are reflected in the bone marrow may be another beneficial application. The reconstruction of orthogonal or arbitrary slices allows an improved assessment of the clinical situation.

The phantom experiments indicate that the passive decoupling during signal excitation is effective but comes at the cost of a reduced Q and signal, at least under the somewhat artificial conditions of the test (Fig. 4). This effect may be attributed to a parasitic resistance of the diodes. In fact, C_1_-type coils without diodes may be beneficial *in vivo* if patient safety is assured.

Whereas the resulting images are intriguing, the topmost challenge for routine application of the presented coils is to improve the stability of the resonance frequency towards loading. A relatively broad resonance line (low Q) is in fact advantageous for this purpose. The stability was already significantly improved by using the silicon putty as a spacer.

Note that we did not attempt to optimize anatomical contrast yet, but chose TR and TE to achieve the shortest scan time. While motion artifacts were not observed in the data acquired, the image quality may be improved further by using motion correction techniques^39^,^40^.

## Conclusions

The presented wireless, intraoral, inductively-coupled coils allowed to acquire dental MRI of a selected region at high resolution like (350 μm)^3^ in a clinically feasible time of approx. 4 min. This is a substantial improvement compared to conventional MRI using large receive coils.

The resolution obtained *in vivo* was comparable to CBCT, the current gold standard for dental imaging. The information obtained, however, was rather complementary, and may aid the diagnostic decision-making process, which is currently being investigated. In particular, gingiva, pulp and cancellous bone were displayed in great detail, as well as the inferior alveolar nerve, its branches and the periodontium.

Whereas the stability of the coils’ resonance frequency requires further improvement, we believe that this development is another important step towards radiation-free, *in vivo* dental imaging. Already now, the coils are suited to monitor longitudinal processes like healing without harmful radiation with unprecedented resolution. In combination with appropriate short-echo time MR sequences like SWIFT^3^, one-stop dental imaging of soft- and hard tissue may become feasible, improving the planning of interventions and thus adding to the overall patient benefit.

## Additional Information

**How to cite this article**: Ludwig, U. *et al.* Dental MRI using wireless intraoral coils. *Sci. Rep.* **6**, 23301; doi: 10.1038/srep23301 (2016).

## Figures and Tables

**Table 1:** Quality factors Q for C_1_, C_2_ and C_3_–type coils measured without load, mounted on phantom P_1_ and *in vivo*.

Coil	Q unloaded	Q test tube	Q *in vivo*
C_1_	335	309	–
C_2_	248	221	–
C_3_	209	190	77

An error of 8% was derived by repetitive measurements *in situ* but is expected to be larger *in vivo* because of a large variability of the coils loading.

**Table 2:** *In-vivo* signal to noise ratio (SNR) of the regions indicated in Fig. 8 for the intraoral coil (IOC) with the loop coil (LC) and LC alone.

Region (indicator)	Fig. 8 panel	SNR IOC + LC	SNR LC	Enhancement
Pulp of first molar (not indicated)	b	31	2	16.6
Marginal gingiva lingual (asterisk)	b	53	6	9.1
Interdental gingiva, papilla (asterisk)	a	57	7	7.6
Interradicular spongiosa (circle)	a	41	16	2.6
Apcial spongiosa (circle)	a	20	19	1.1
Signal void in between loops (circle)	b	3	17	0.2
Standard deviation of noise (circle)	b	4.5	7.4	–

The enhancement is the ratio of the SNR acquired with IOC+LC divided by the SNR of LC. The error was estimated to 10%.
